# Potential clinical application of microRNAs in bladder cancer

**DOI:** 10.7555/JBR.37.20230245

**Published:** 2024-05-29

**Authors:** Pei Wang, Xiaowei Wei, Xiaojun Qu, Yefei Zhu

**Affiliations:** 1 Laboratory Medicine Center, the Second Affiliated Hospital of Nanjing Medical University, Nanjing, Jiangsu 210011, China; 2 School of Biological Science and Medical Engineering, Southeast University, Nanjing, Jiangsu 210096, China

**Keywords:** bladder cancer, microRNAs, biomarker, diagnosis, detection method

## Abstract

Bladder cancer (BC) is the tenth most prevalent malignancy globally, presenting significant clinical and societal challenges because of its high incidence, rapid progression, and frequent recurrence. Presently, cystoscopy and urine cytology serve as the established diagnostic methods for BC. However, their efficacy is limited by their invasive nature and low sensitivity. Therefore, the development of highly specific biomarkers and effective non-invasive detection strategies is imperative for achieving a precise and timely diagnosis of BC, as well as for facilitating an optimal tumor treatment and an improved prognosis. microRNAs (miRNAs), short noncoding RNA molecules spanning around 20–25 nucleotides, are implicated in the regulation of diverse carcinogenic pathways. Substantially altered miRNAs form robust functional regulatory networks that exert a notable influence on the tumorigenesis and progression of BC. Investigations into aberrant miRNAs derived from blood, urine, or extracellular vesicles indicate their potential roles as diagnostic biomarkers and prognostic indicators in BC, enabling miRNAs to monitor the progression and predict the recurrence of the disease. Simultaneously, the investigation centered on miRNA as a potential therapeutic agent presents a novel approach for the treatment of BC. This review comprehensively analyzes biological roles of miRNAs in tumorigenesis and progression, and systematically summarizes their potential as diagnostic and prognostic biomarkers, as well as therapeutic targets for BC. Additionally, we evaluate the progress made in laboratory techniques within this field and discuss the prospects.

## Introduction

Based on the 2021 global cancer statistics, bladder cancer (BC) is the tenth most prevalent malignancy worldwide, with approximately 573000 newly diagnosed cases and 213000 deaths^[1]^. The incidence of BC is three times higher in males than in females, and it will continue to grow as the global population expands and ages. BC is a highly heterogeneous disease, with over 90% of the cases originating in the urothelium and other types including squamous cell carcinoma, adenocarcinoma, or neuroendocrine tumors^[2]^. In newly diagnosed BC, 75% were non-muscle-invasive BCs (NMIBCs) confined to the mucosa or submucosal connective tissues, and 25% were muscle-invasive BCs (MIBCs) that spread to or through the detrusor muscles^[3]^. Despite the favorable survival rates in patients with NMIBC, its susceptibility to the recurrence is remarkably high (> 50%), with 10%–25% of the cases progressing to MIBC and a poor 5-year overall survival of < 50%^[3–4]^. Consequently, a timely and precise diagnosis of BC is of paramount importance in determining patient prognosis.

Cystoscopy is the prevailing diagnostic approach for BC, but with substantial invasiveness, frequently leading to severe complications in the patients. Moreover, the routine use of cystoscopy for monitoring tumor progression or recurrence substantially amplifies the financial strain on the patients and concurrently diminishes patient adherence^[5]^. Non-invasive diagnostic techniques, such as urine cytology, exhibit a reduced sensitivity towards early and low-grade BC, and negative outcomes do not entirely rule out the presence of tumors^[6]^. The Food and Drug Administration has approved various biomarker tests for BC diagnosis, such as NMP22 BC, NMP22 BladderChek, BTA Stat, BTA TRAK, UroVysion, and uCyt+/ImmunoCyt. However, these methods have been demonstrated to have limited sensitivities in detecting early low-grade and recurrent BC, and therefore they cannot serve as a substitute for the conventional clinical diagnosis strategy^[7]^. Hence, there is an immediate need to identify new biomarkers that have a high degree of specificity and sensitivity to facilitate the non-invasive detection of BC.

microRNAs (miRNAs), short non-coding RNAs consisting of approximately 20–25 nucleotides, have been extensively studied and found to exhibit abnormal expression patterns in various types of human cancers^[8]^. These miRNAs play a crucial role in the initiation and progression of cancers. The capacity of miRNAs to interact with various RNA-binding proteins or to exist within extracellular vesicles (EVs) allows them to remain stable in challenging conditions, such as urine, without being degraded by RNase^[9]^. Consequently, miRNAs have attracted a significant interest as emerging biomarkers for urinary tumors, particularly BC. In this review, we provide an overview of biological attributes of miRNAs and elucidate their significance in carcinogenesis and progression. Specifically, we present a comprehensive analysis of miRNAs as potential diagnostic and prognostic biomarkers, and therapeutic targets for BC, by using blood, urine, and EV specimens. Furthermore, we summarize the advances in diagnostic techniques of miRNAs.

## Biological properties of miRNAs

**Figure 1:**
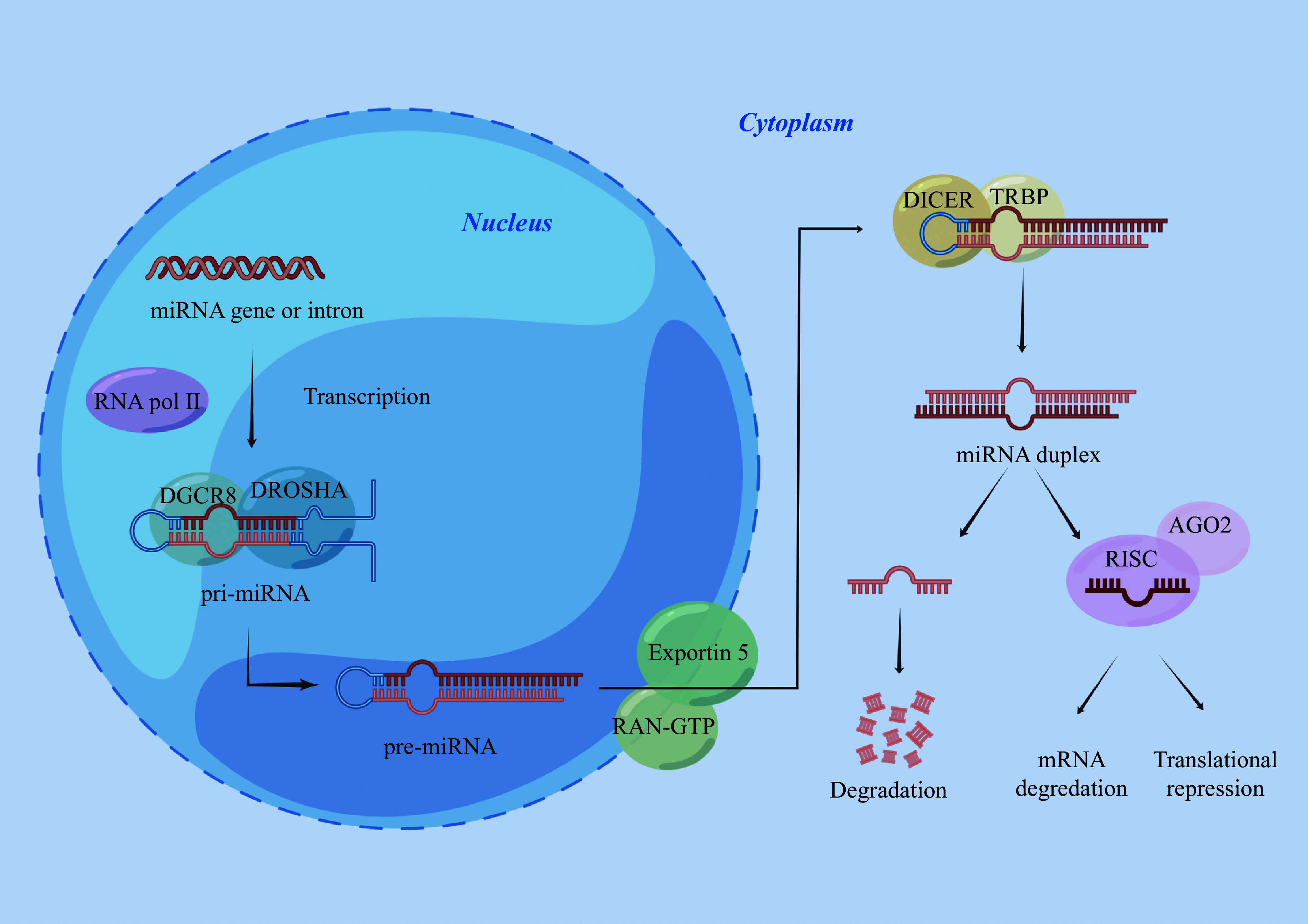
A schematic diagram of miRNA biogenesis.

The fundamental characteristics of miRNA biogenesis are illustrated in ***Fig. 1***. Transcription initiation of the miRNA coding sequence is facilitated by RNA polymerase Ⅱ in the nucleus, resulting in the formation of the primary miRNAs^[10]^. Subsequently, the primary miRNAs are cleaved by RNA polymerase Ⅲ DROSHA and the RNA-binding protein DGCR8 into precursor miRNAs with a hairpin loop secondary structure^[11]^. After the initial cleavage, the precursor miRNAs are transported from the nucleus into the cytoplasm under the action of the transporter exportin 5, and then further cleaved by another RNase type Ⅲ enzyme called DICER and the cofactor TAR-RNA-binding protein (TRBP) to produce the miRNA double-stranded complex^[12]^. The guide strand, in conjunction with the argonaute 2 (AGO2) protein, constitutes the RNA-induced silencing complex (RISC), and the other companion strand is usually degraded^[13]^. Once biosynthesized, the structural domains within the AGO2 protein undergo conformational alterations, leading to the binding of the guide strand to the complementary sequence of the target mRNA^[14]^. This binding subsequently directs the RISC toward its intended target, facilitating two distinct modes of gene silencing: mRNA degradation and translation inhibition^[15]^.

## The role of miRNAs in carcinogenesis and progression

miRNAs play a crucial role in regulating gene expression at the post-transcriptional level, targeting numerous downstream genes involved in various physiological processes^[16]^. Consequently, aberrant miRNA expression may contribute to the development and progression of diverse diseases, including cancers. The association between miRNAs and cancers was initially established two decades ago with the identification of miR-15/16 as a tumor suppressor in B-cell chronic lymphocytic leukemias^[17]^. It is noteworthy that miRNAs regulate the expression of more than 60% of human genes^[18]^. Over 50% of the miRNA genes are located within cancer-associated regions of the human genome, which are susceptible to loss or rearrangement^[8]^. The intricate molecular patterns of miRNA expression variations in cancers are governed by various mechanisms, encompassing miRNA site deletion or amplification, miRNA gene mutation, epigenetic and transcriptional regulation, post-transcriptional modifications, and the control of miRNA dysregulation^[19]^.

Some miRNAs modulate the signaling pathways of target genes in a manner similar to oncogenes or tumor suppressor genes, and may affect cancer self-renewal, spheroid formation, cancer recurrence, migration, invasion, and chemical and radiotherapy resistance^[18]^. For example, the upregulated oncogene miR-4739 promotes epithelial-mesenchymal transformation (EMT) and angiogenesis in lung cancer by activating the Wnt/β-catenin signaling^[20]^. The down-regulated tumor suppressor miR-2392 directly targets Jagged 2 (JAG2) overexpression and promotes the malignant progression of liver cancer^[21]^. The specific action modes of these miRNAs are largely dependent on the tissue or organ specificity of the target genes, suggesting the potential to identify characteristic miRNAs in various types of cancers. In addition, the interaction between miRNAs and target genes is influenced by various factors, including the subcellular location of miRNAs, the abundance of miRNAs and target genes, and the binding affinities between them^[22]^.

**Figure 2:**
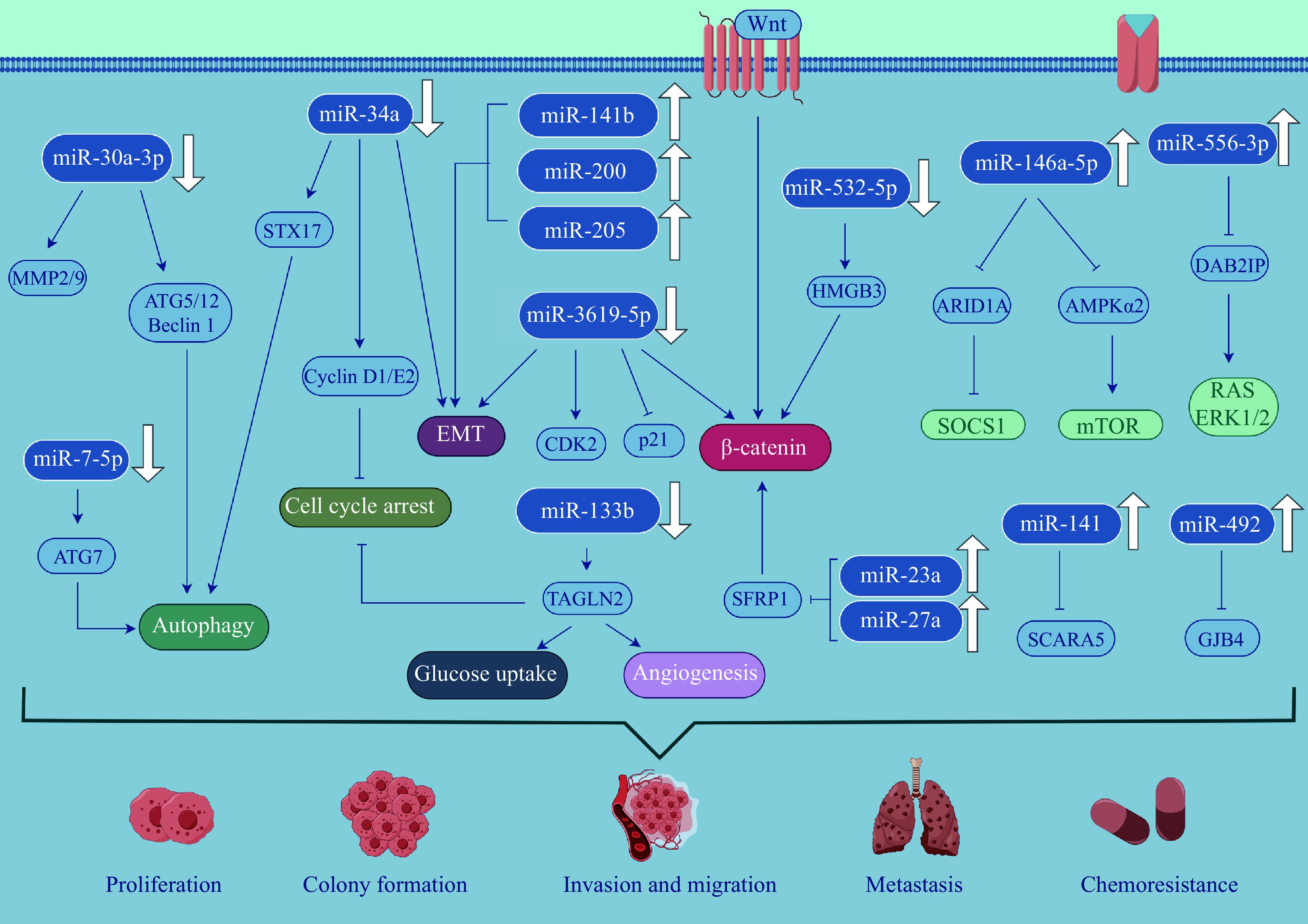
miRNAs play significant roles in the carcinogenesis and progression of bladder cancer.

There is some accumulating evidence indicating that the differential expression of miRNAs plays a significant role in the carcinogenesis and progression of BC (***Fig. 2***). miR-141 promotes BC cell proliferation, invasion, and migration by targeting down-regulation of scavenger receptor class A member 5 (SCARA5)^[23]^. miR-556-3p enhances RAS GTPase activity and extracellular signal-related kinases 1 and 2 (ERK1/2) phosphorylation by targeting the down-regulation of DAB2 interacting protein (DAB2IP), and stimulates the proliferation, invasion, migration, and colony formation of BC cells^[24]^. miR-492 promotes proliferation and metastasis and inhibits apoptosis of BC cells by targeting gap junction protein beta 4 (GJB4)^[25]^. Both miR-23a and miR-27a target secreted frizzled-related protein 1 (SFRP1) to negatively regulate Wnt/β-catenin, mediating BC cell proliferation, migration, invasion, and sensitivity to radiation and cisplatin therapy^[26]^. miR-532-5p inhibits the proliferation and invasion of BC cells by targeting high-mobility group protein B3 (HMGB3) and the Wnt/β-catenin signaling, but its expression in BC is down-regulated^[27]^. miR-30a-3p targets the oncogenes matrix metalloproteinase 2 (MMP2) and MMP9 and inhibits the growth and invasion of BC cells^[28]^. miR-3619-5p interferes with BC cell growth and metastasis by targeting β-catenin and cyclin-dependent kinase 2 (CDK2) and activating p21^[29]^. miR-34a inhibits cell proliferation, triggers G1/G2 cell cycle arrest, and promotes chemical sensitivity by targeting cyclin D1/E2, EMT, and syntaxin 17 (STX17)^[30]^. miR-7-5p affects BC cell migration, invasion, autophagy, and chemical resistance by regulating the autophagy-related 7 (ATG7)^[31]^. The miR-133b/transgelin 2/cell cycle pathway axis controls BC cell proliferation, glucose uptake, invasion, angiogenesis, colony formation, and gemcitabine chemical sensitivity^[32]^.

## Potential clinical application

### miRNAs and BC diagnosis/prognosis

The identification of potential biomarkers with a high potency is crucial for the precise and prompt diagnosis of BC, which is a vital requirement for the effective cancer treatment and the enhancement of patient prognosis. Investigations on mRNAs as biomarkers have paved the way for the use of miRNAs in clinical diagnosis. Some empirical evidence has demonstrated that miRNAs exhibit a greater accuracy than mRNAs in distinguishing poorly differentiated cancer, thereby establishing miRNAs as highly promising biomarker candidates for early cancer diagnosis^[33–34]^. For example, miR-371a-3p is a novel serum marker for testicular germ cell tumors, which is far more sensitive and specific than classical serum markers^[35]^. Moreover, monitoring the recurrence and progression from NMIBC to MIBC or metastasis holds an equal importance. Nevertheless, the existing monitoring indicators, including tumor grade, tumor size, and multifocality, are constrained in their ability to facilitate reliable risk-adjusted treatment decisions because of their inability to offer real-time and precise clinical information^[36]^.

#### miRNAs in blood

Numerous studies have demonstrated that diagnostic accuracy for BC cases is enhanced when multiple miRNAs are used in combination, compared with using individual miRNAs alone. A recent study identified differential expression of five miRNAs in serum specimens obtained from both BC patients and healthy individuals; by constructing a diagnostic model incorporating miR-27b-3p, miR-381-3p, and miR-451a, it was determined that this combination had a favorable diagnostic capability for early BC^[37]^. However, this study solely examined the levels of miRNA expression in serum and did not proceed to confirm the consistency of their expression in histological contexts. Conversely, another study employed microarrays to profile plasma miRNAs and subsequently validated the findings through reverse transcription-quantitative polymerase chain reaction (RT-qPCR); eight miRNAs with differential expression levels were identified, especially for the combination of miR-497 and miR-663b that proved to be effective biomarkers for distinguishing between BC patients and healthy individuals; furthermore, the investigators found that the expression levels of miR-663b in tumor tissues showed an unexpected and significant reduction, contrary to that observed in plasma^[38]^.

In a comprehensive study using microarrays, a diagnostic combination of seven miRNAs showed an exceptional accuracy in distinguishing BC from non-cancerous conditions and other tumor types; this combination used in BC patients with low-grade stage and < pT2 stage demonstrated a remarkable sensitivity (92.7% and 94.7%, respectively), surpassing the effectiveness of urine cytology as indicated by a significantly higher area under the curve (AUC)^[39]^. However, it should be noted that this combination fails to differentiate between NMIBC and MIBC, which is crucial information for determining the necessity of cystectomy.

In a study of serum miRNAs and BC diagnosis, Wang *et al*^[40]^ discovered a significant up-regulation of miR-17–92 clusters in BC tissues, cell lines, and serum samples from BC patients, compared with normal controls. The diagnostic efficacy of miR-92a-3p, miR-17-5p, and miR-20a-5p used in a three-miRNA diagnostic model resulted in an increased AUC of 0.969. In contrast to microarray screening, however, high-throughput sequencing eliminates some potential contamination of other small RNA and DNA fragments. For example, Jiang *et al*^[41]^ employed MiSeq to identify 26 differentially expressed miRNAs in BC, and found that the AUC for the combination of six miRNAs used for early BC diagnosis in patients with Ta and T1 stages was significantly higher (0.841), compared with that of urine cytology (0.645)^[41]^. In a study conducted by Li *et al*^[42]^, a combination of four miRNAs was identified for BC diagnosis in the serum, demonstrating an exceptional diagnostic power with an AUC of 0.985.

Additionally, it was found that the expression of plasma miR-10a-5p showed a promising performance in distinguishing BC patients from healthy individuals before surgery, as well as differentiating NMIBC from MIBC; the elevated expression of miR-10a-5p was found to be a significant predictor of unfavorable overall survival and progression-free survival outcomes in individuals diagnosed with BC; furthermore, miR-10a-5p was implicated in the facilitation of BC cell proliferation, migration, and invasion^[43]^. Similarly, high levels of miR-19a expression in the plasma of BC patients were found to be associated with a more aggressive tumor phenotype, indicating a bleak prognosis^[44]^.

Furthermore, investigations of the correlation between serum miRNAs and BC recurrence suggest that specific miRNAs hold the promise as biomarkers for predicting BC recurrence and improving prognosis. Higher expression of miR-152 as well as lower expression of both miR-3187-3p and miR-27a-3p are associated with a poorer prognosis of BC patients with characteristics of advanced clinical stage, higher tumor grade, and positive lymph node metastasis. Specifically, NMIBC patients with high miR-152 expression and low miR-3187-3p expression had a poor relapse-free survival, miR-152 was independently associated with tumor recurrence in NMIBC^[41]^, and miR-20a-5p was also associated with BC recurrence^[40]^. In addition, the expression levels of miR-210 were associated with the staging and grading of BC, and the expression of serum miR-210 was significantly decreased in postoperative paired specimens, but increased in most patients with recurrent BC^[45]^ (***Table 1***).

**Table 1:** Diagnostic and prognostic role of blood miRNAs in bladder cancer

miRNAs	Source	BC/control (*n*)	Target genes/mechanism	Biological function	Remarks	References
miR-492↑	Serum	23/23	*GJB4*	Diagnosis; prognosis	AUC=0.86; progress	[25]
miR-27b-3p↑ miR-381-3p↓ miR-451a↓	Serum	112/112	*SMAD4*, *FOXO1*	Diagnosis	3-miRs: AUC=0.894, sensitivity=86.90%, specificity=77.38%	[37]
miR-497↑ miR-663b↑	Plasma	165/175	–	Diagnosis	2-miRs: AUC=0.711, sensitivity=69.7%, specificity=69.6%	[38]
miR-6087↓ miR-6724-5p↑ miR-3960↓ miR-1343-5p↓ miR-1185-1-3p↑ miR-6831-5p↑ miR-4695-5p^a^	Serum	392/580	–	Diagnosis	7-miRs: AUC=0.97 sensitivity=95%, specificity=87%	[39]
miR-20a-5p↑ miR-92a-3p↑ miR-17-5p↑	Serum	74/90	–	Diagnosis	AUC=0.806; 0.902; 0.845; 3-miRs: AUC=0.969, sensitivity=90.36%, specificity=94.44%	[40]
miR-20a-5p↑	Serum	74/90	–	Prognosis	Recurrence	[40]
miR-152↑ miR-148b-3p↑ miR-3187-3p↓ miR-15b-5p↓ miR-27a-3p↓ miR-30a-5p↓	Serum	250/240	–	Diagnosis	6-miRs: AUC=0.899, sensitivity=80.00%, specificity=89.09%	[41]
miR-152↑ miR-3187-3p↓	Serum	250/240	–	Prognosis	Recurrence	[41]
miR-182-5p↑ miR-196a-5p↑ miR-124-3p↓ miR-34a-5p↓	Serum	132/122	*MAPK*	Diagnosis	4-miRs: AUC=0.985, sensitivity=98.78%, specificity=93.75%	[42]
miR-10a-5p↑	Plasma	208/36	–	Diagnosis; prognosis	Diagnosis: AUC=0.815, sensitivity=79.5%, specificity=65.6%; progress: AUC=0.785, sensitivity=75.0%, specificity=64.2%	[43]
miR-19a↑	Plasma	50/50	*PTEN*	Prognosis	Progress	[44]
miR-210↑	Serum	168/177	–	Prognosis	Recurrence	[45]
^a^The expression level of miR-497-5p was not significantly altered between BC and controls. En-dash (–) indicates no data available. Abbreviations: BC, bladder cancer; miR, miRNA; AUC, area under the curve.						

#### miRNAs in urine

Intrinsic properties of the urine, when in direct proximity to tumor tissue within the bladder wall, render it an indispensable reservoir of biomarkers. In a recent study, a novel diagnostic system for BC, named BlaDimiR, was developed^[46]^. The investigators determined that the miR-145/miR-182 ratio exhibited the highest suitability as a miRNA indicator for predicting the existence of BC, demonstrating a specificity comparable to cytological detection and surpassing cystoscopy in terms of sensitivity. BlaDimiR offers several advantages, including the minimal effect on hematuria, high diagnostic accuracy in identifying low- and high-risk tumors, and the independence of positive results from previous tumor occurrences^[46]^.

In their study, Erdmann *et al*^[47]^ reported that a combination of four miRNAs (miR-125b, miR-145, miR-183, and miR-221) from urine sediment along with voided urine cytology (VUC) demonstrated an optimal diagnostic potential for non-invasive detection of BC. This combination showed an increase in negative predictive value (NPV) and sensitivity by nearly 8% and a diagnostic capacity of 0.88. Another combination of miR-210, miR-10b, and miR-29c from urine sediment along with VUC increased the sensitivity of detection to 95.2%^[48]^. In addition, Eissa *et al*^[49]^ and Yamada *et al*^[50]^ improved diagnostic sensitivity and overall accuracy through a combined analysis of VUC and miR-96.

Because hematuria is a key symptom of BC, it is also important to distinguish the cancer-specific miRNAs from the bladder-derived miRNAs to reduce the unnecessary cystoscopy for BC evaluation in hematuria patients. The ratio of miR-6124 to miR-4511 in the urine of BC patients was significantly superior to that in the urine of healthy subjects, hematuria, and pyuria patients (AUC = 0.865, 0.888, and 0.907, respectively), and both the two miRNAs could discriminate NMIBC patients and MIBC patients from healthy controls (AUC = 0.803 and 0.761, respectively)^[51]^.

Lin *et al*^[52]^ performed the "next-generation" sequencing (NGS) technology analysis on urine specimens from 10 BC patients and 10 healthy subjects to generate a miRNA profile, and found 50 differentially expressed miRNAs. The investigators screened five up-regulated miRNAs (let-7b-5p, miR-146-5p, miR-149-5p, miR-423-5p, and miR-193a-5p) for validation in the database and found that these miRNAs were also significantly expressed in BC tissues. They further validated their results in the urine of 70 BC patients and 90 healthy subjects and found that four miRNAs other than miR-193a-5p were also upregulated in BC urine. Unfortunately, the authors did not give diagnostic AUC values and prognostic abilities of these miRNAs in urine.

Cavallari *et al*^[53]^ noted that the use of small nuclear/nucleolar RNAs, such as *RNU6*, *RNU44*, and *RNU48*, as normalizers was controversial, because of their different lengths, processing, and subcellular compartmentalization, compared with miRNAs. Instead, they selected miR-125b with a lower standard deviation and a higher statistical test performance as a suitable normalizer. In their large-scale study, after excluding the miRNAs affected by confounding factors, such as hematuria and urine specific gravity, they found that the elevated levels of six miRNAs in urine were associated with a poorer progression-free survival. Using a two-step decision tree based on the urine levels of miR-34a-5p, miR-200a-3p, and miR-193a-5p, BC patients could be classified as high- or low-risk of death with a sensitivity of 0.844, specificity of 0.806, and accuracy of 0.825.

Notably, the let-7c cluster (*i.e.*, let-7c, miR-99a, and miR-125b) is a reliable biomarker (AUC = 0.80) that distinguishes high-grade NMIBC T1 and Ta. It was shown that upregulation of the let-7c family also similarly affected progression-free survival, thus suggesting that the expression of these miRNAs may improve the prognostic estimation and identify patients at progression risk^[54]^. Sapre *et al*^[55]^, by focusing on miRNAs for BC monitoring, found that a combination of six-miRNA combination holds a significant promise for monitoring tumor recurrence (***Table 2***).

**Table 2:** Diagnostic/prognostic role of urinary miRNAs in bladder cancer

miRNAs	BC/control (*n*)	Target genes/mechanism	Biological function	Remarks	References
miR-145↓ miR-182↑	119/24	–	Diagnosis	The ratio AUC=0.97, sensitivity=92%, specificity=92%	[46]
miR-125b↓ miR-145↓ miR-183 ↑ miR-221↓	104/46	–	Diagnosis	4-miRs+VUC: sensitivity=84.6%, NPV=73.3%, accuracy=88.0%	[47]
miR-210↑ miR-10b↑ miR-29c↑	188/180	Focal adhesion, *MAPK*, Wnt signaling	Diagnosis	3-miRs+VUC: AUC=0.875, sensitivity=95.2%, specificity=79.4%, PPV= 86.9%, NPV=94%	[48]
miR-96↑	94/90	–	Diagnosis	Combination with VUC: AUC=0.83, sensitivity=79.8%, specificity=86.7%, PPV=86.2%, NPV=80.4%	[49]
miR-96↑ miR-183↑	100/74	*BAX*, *COL18A1*, *ADAMTSL4*, *TRAF5*, *DUSP1*, *PACS2*, *APH1A*, *VEGFA*, *PERP*, *GADD45B*	Diagnosis	Combination of miR-96 with VUC=78.2%	[50]
miR-6124↑ miR-4511↑	326/227	–	Diagnosis	The ratio AUC=0.810, sensitivity=78.5%, specificity=70.9%	[51]
miR-29a-3p↑ miR-34a-5p↑ miR-193a-5p↑ miR-200c-3p↑ miR-205-5p ↑ miR-532-5p↑	63/37	–	Prognosis	Progression-free survival rate	[53]
let-7c↑ miR-99a↑ miR-125b↑	57/20	–	Prognosis	Progression-free survival	[54]
miR-16↑ miR-200c↑ miR-205↑ miR-21↑ miR-221↑ miR-34a↑	110/21	*PCTH1*, *FOXL1*, *BCL2*, *CCND2*, *ZEB2*	Prognosis	Recurrence: AUC=0.74, sensitivity=88%, specificity=48%	[55]
miR-34a-5p↑ miR-205-3p↑ miR-210-3p↑	81/66	–	Diagnosis	3-miRs+SERS: AUC=0.92, superior either to miRs (AUC=0.84) or SERS data (AUC=0.84) individually	[104]
En-dash (–) indicates no data available. Abbreviations: BC, bladder cancer; AUC, area under the curve; SERS, surface enhanced Raman spectroscopy; VUC, voided urine cytology; NPV, negative predictive value; PPV, positive predictive value.					

#### miRNAs in EVs

**Figure 3:**
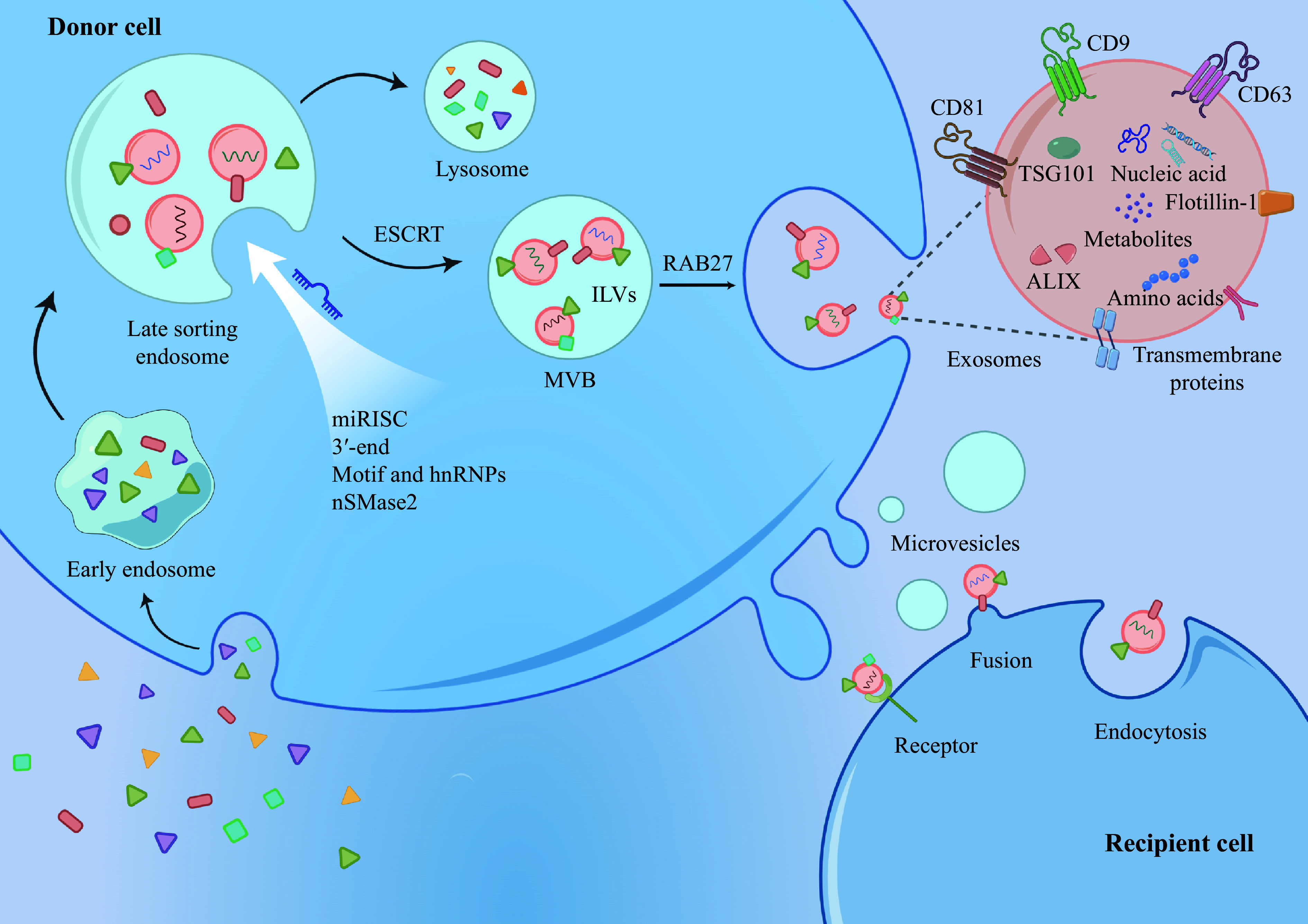
A schematic diagram of exosome secretion and uptake.

As membrane vesicles rich in biomolecular substances, such as nucleic acids, proteins, amino acids, and metabolites, EVs play an essential role in intercellular communication^[56]^. Based on their production and secretion, EVs can be roughly classified into three main categories, including microvesicles (200–2000 nm in diameter), apoptotic bodies (500–5000 nm in diameter), and exosomes (30–200 nm in diameter)^[57]^. The plasma membrane vesicles form directly from microvesicles or apoptotic bodies outward, while exosomes are produced in the endosomal system (***Fig. 3***). Early endosomes formed by plasma membrane endocytosis form multivesicular bodies (MVBs) containing intraluminal vesicles (ILVs). MVBs are finally fused and degraded directly with lysosomes or are transported to the plasma membrane to release exosomes^[58]^. Several proteins are involved in exosome biogenesis, including the endosomal sorting complex required for transport (ESCRT), the direct regulator of MVBs transport to the plasma membrane (RAB27), the quaternary transmembrane proteins (CD9, CD81, and CD63), tumor susceptibility gene 101 (TSG101), apoptosis-linked gene 2 interacting protein X (ALIX), and flotillin^[59]^. Recipient cells take up exosomes by fusion with the vesicle membrane, by ligand-mediated pathways on the receptor, or by the endocytosis^[56,60]^.

There is still a lack of consensus on the nomenclature of exosomes and other vesicles, and this review follows the use of the term "extracellular vesicles" as a generic term for isolating and studying vesicles by the International Society for Extracellular Vesicles. According to the existing studies, there are four potential pathways to assemble miRNA into EVs: the neutral sphingomyelinase 2 (nSMase2)-dependent pathway, the miRNA motif and heterogeneous nuclear ribonucleoproteins (hnRNPs)-dependent pathway, the 3′-end of the miRNA sequence-dependent pathway, and the miRNA-induced silencing complex (miRISC)-related pathway^[61]^. EVs are found in most fluids with specific expression patterns from their parent cells, and the miRNAs they contain have unique expression profiles, suggesting that EV miRNAs provide a critical basis for valuable non-invasive cancer diagnosis and prognosis^[62]^.

To explore clinical efficacy of the biological specimen types for further BC diagnosis, Armstrong *et al*^[63]^ adopted the NanoString miRNA assay and droplet digital PCR (dPCR) for validation, and found that a large number of miRNAs up-regulated in BC tissues were recognized in urine EVs and white blood cells of the same patient, but not in plasma. Among all biological specimens, the expression of miR-4454 and miR-21 was the most common, while the expression of miR-720/3007a, miR-205, miR-200c-3p, and miR-29b-3p was commonly seen in tumors/urine. This study also found no association between the hemolysis replacement indicator (miR-451a) and miR-4454 or miR-720/3007a, enhancing the sensitivity of urine EV miRNAs as potential sources of specific diagnostic biomarkers^[63]^. It was also reported that AUCs for miR-93-5p and miR-516a-5p in urinary EVs were statistically significant (0.838 and 0.790, respectively), which were higher than that of urine cytology (0.63) in BC diagnosis; however, there was no significant difference between the two miRNA combinations and a single miRNA in their use of BC diagnosis prediction^[64]^. The combination of three miRNAs (miR-139-5p, miR-136-3p, and miR-19b1-5p) in urine EVs may improve the accuracy of differentiating between cancer and non-cancer patients (AUC = 0.903), and the same model may also distinguish the low-risk group from the healthy group with 93% sensitivity and 97% specificity (AUC = 0.976)^[65]^. Another study found that a low expression of miR-185-5p and miR-106a-5p as well as a high expression of miR-10b-5p detected by NGS in plasma EVs were associated with a short survival in BC patients, suggesting a poor prognosis^[66]^.

The disparities of EV miRNA expression between NMIBC and MIBC in previous studies underscore the potential significance of EV biomarkers in the surveillance and diagnosis of BC patients. Notably, the upregulation of miR-93-5p exhibited a greater prominence in MIBC than in NMIBC, potentially through the targeted inhibition of B-cell translocation gene 2 (BTG2) to promote cancer cell proliferation, migration, and invasion^[64]^. Compared with both NMIBC and healthy groups, the expression of miR-139-5p and miR-19b1-5p in the MIBC metastasis group was downregulated^[65]^. In a study of analyzing the differential expression of miRNAs in urinary EVs, it was found that miR-146b-5p and miR-155-5p were highly expressed in MIBC^[67]^. miR-375 may be used as a biomarker for high-grade BC, while miR-146a may identify patients with low-grade BC; furthermore, high expression of miR-146a in low-grade patients appeared to be associated with non-recurrence^[68]^. Both miR-451a and miR-486-5p were highly expressed in preoperative urinary EVs of T1 patients, which may be potential biomarkers for relapse-free survival of BC patients in the early stage^[69]^ (***Table 3***).

**Table 3:** Diagnostic/prognostic role of extracellular vesicle miRNAs in bladder cancer

miRNAs	Source	BC/control (*n*)	Target genes/mechanism	Biological function	Remarks	References
miR-93-5p↑ miR-516a-5p↑	Urine	65/55	*BTG2*	Diagnosis	AUC=0.867, sensitivity=85.2%, specificity=82.4%	[64]
miR-93-5p↑	Urine	65/55	*BTG2*	Prognosis	Progress	[64]
miR-139-5p↓ miR-136-3p↑ miR-19b1-5p↓	Urine	59/34	–	Diagnosis	AUC=0.903, sensitivity=80%, specificity=88.2%	[65]
miR-139-5p↓ miR-19b1-5p↓	Urine	59/34	*MMP11*, *PTEN*	Prognosis	Progress	[65]
miR-185-5p↓ miR-106a-5p↓ miR-10b-5p↑	Plasma	47/46	–	Prognosis	Survival rate	[66]
miR138-5p↓ miR144-5p↓ miR200a-3↓ miR146b-5p↑ miR155-5p↑	Urine	37	–	Prognosis	Progress	[67]
miR-146a↑ miR-375↓	Urine	34/9	–	Prognosis	Progress and recurrence	[68]
miR-451a↑ miR-486-5p↑	Urine	41/15	*CAB39*, *CDKN2D* *HAT1*, *FOXO*	Prognosis	Recurrence-free survival	[69]
En-dash (–) indicates no data available. Abbreviations: BC, bladder cancer; AUC, area under the curve.						

### miRNAs and BC treatment

Currently, the primary treatment for BC is surgery, but a quarter of patients still have an unsatisfying prognosis^[70]^. The identification of aberrant miRNA expression and oncogenic or tumor-suppressive targets regulated by miRNAs is the primary prerequisite for the development of new miRNA-based therapeutic regimens. Because of the tissue specificity of miRNA regulation, a single miRNA can target multiple mRNAs and a single mRNA can be targeted by multiple miRNAs^[71–72]^. Given this complexity, the development of miRNA-based therapeutics for BC is even more advantageous and attractive. To reduce recurrence rates and improve survival in BC patients, the combination chemotherapy is a promising therapy option. However, because of the high somatic mutagenicity and heterogeneity of BC tumors, they are prone to drug resistance during therapy, which seriously reduces survival expectations of the patients^[73]^. Furthermore, miRNAs regulate drug sensitivity in tumor cells by targeting drug-resistant genes or influencing pathways associated with cell proliferation, cell cycle, apoptosis, cancer stem cells, and EMT^[74–75]^. ***Table 4*** summarizes the miRNAs involved in BC chemoresistance and related targets and functions^[26,28, 30–32,76–83]^.

**Table 4:** Summary of miRNAs in BC chemotherapy drugs

miRNAs	Target genes/mechanism	Remarks	References
miR-23a	*SFRP1*, Wnt signaling	Associated with radiotherapy and cisplatin therapy	[26]
miR-27a	*SLC7A11*, *SFRP1*, Wnt signaling, *RUNX1*	Expression reduced in cisplatin-resistant BC, *SLC7A11* up-regulated, and GSH biosynthesis increased, promoting cisplatin resistance^[81]^; associated with radiotherapy and cisplatin therapy^[26]^; rs11671784 A replaced by G, reducing the chemical sensitivity^[80]^	[26,80–81]
miR-30a-3p	*ATG5*, *ATG12*, *BECN1*	Inhibiting muscle invasion combined with cisplatin and enhancing anti-tumor effect	[28]
miR-34a	*CD44*, *CDK6*, *SRT1*, *TCF1*, *LEF1*, *STX17*	Overexpression in BC cells enhanced the chemosensitivity of cisplatin, doxorubicin, epirubicin, and mitomycin C	[30,77–79]
miR-7-5p	*ATG7*	Upregulation of miR-7-5p inhibited the invasive characteristics and promoted the chemosensitivity	[31]
miR-133b	*TAGLN2*	miR-133b inhibited glucose uptake, invasion, angiogenesis, and enhanced gemcitabine chemosensitivity of BC cells *in vitro*	[32]
miR-424	*UNC5B*, *SIRT4*	Promotion of cisplatin resistance by downregulation of *UNC5B* and *SIRT4*	[76]
miR-99a-5p	*SMARCD1*	Tumor suppressor, downregulating *SMARCD1* to induce cellular senescence in gemcitabine-resistant BC	[82]
miR-146a-5p	*ARIDIA*, *AMPK2*	miR-146a-5p derived from cancer-associated fibroblasts promoted stemness and enhanced chemoresistance	[83]
Abbreviation: BC, bladder cancer.			

In addition, several miRNA-based BC therapeutic approaches currently under development are summarized as follows: (1) miRNA antagonists. The antagonist of oncogene miR-708-3p down-regulates caspase-2 levels, promotes apoptosis, and inhibits BC growth^[84]^. The application of miR-146a-5p antagonists is a promising therapeutic strategy for recurrent BC^[85]^. (2) miRNA mimics. miR-139-5p in bone marrow mesenchymal stem cell-derived EVs may delay the occurrence of BC^[86]^. After transfection with miR-133b mimics, BC proliferation may be inhibited by upregulating dual-specificity protein phosphatase 1^[87]^. (3) Epigenetic drugs. The regulation of DNA methyltransferase 3B (DNMT3B) mediates miR-124-3p, inhibits proliferation, migration, and invasion, and promotes apoptosis of BC cells^[88]^. (4) miRNA delivery strategy. Exosomes derived from adipose mesenchymal stem cells penetrate BC tumor tissues and successfully deliver miR-138-5p to inhibit tumor growth^[89]^. Mesoporous silica nanoparticles can be used in the delivery of antitumor factors miR-34a and miR-200c^[90–91]^. (5) Radiation sensitizers. miR-1246 may enhance the radiotherapy sensitivity in BC cells by targeting and inhibiting *TP53* gene translation^[92]^.

## Laboratory diagnostic techniques for miRNAs

### RT-qPCR and dPCR

**Figure 4:**
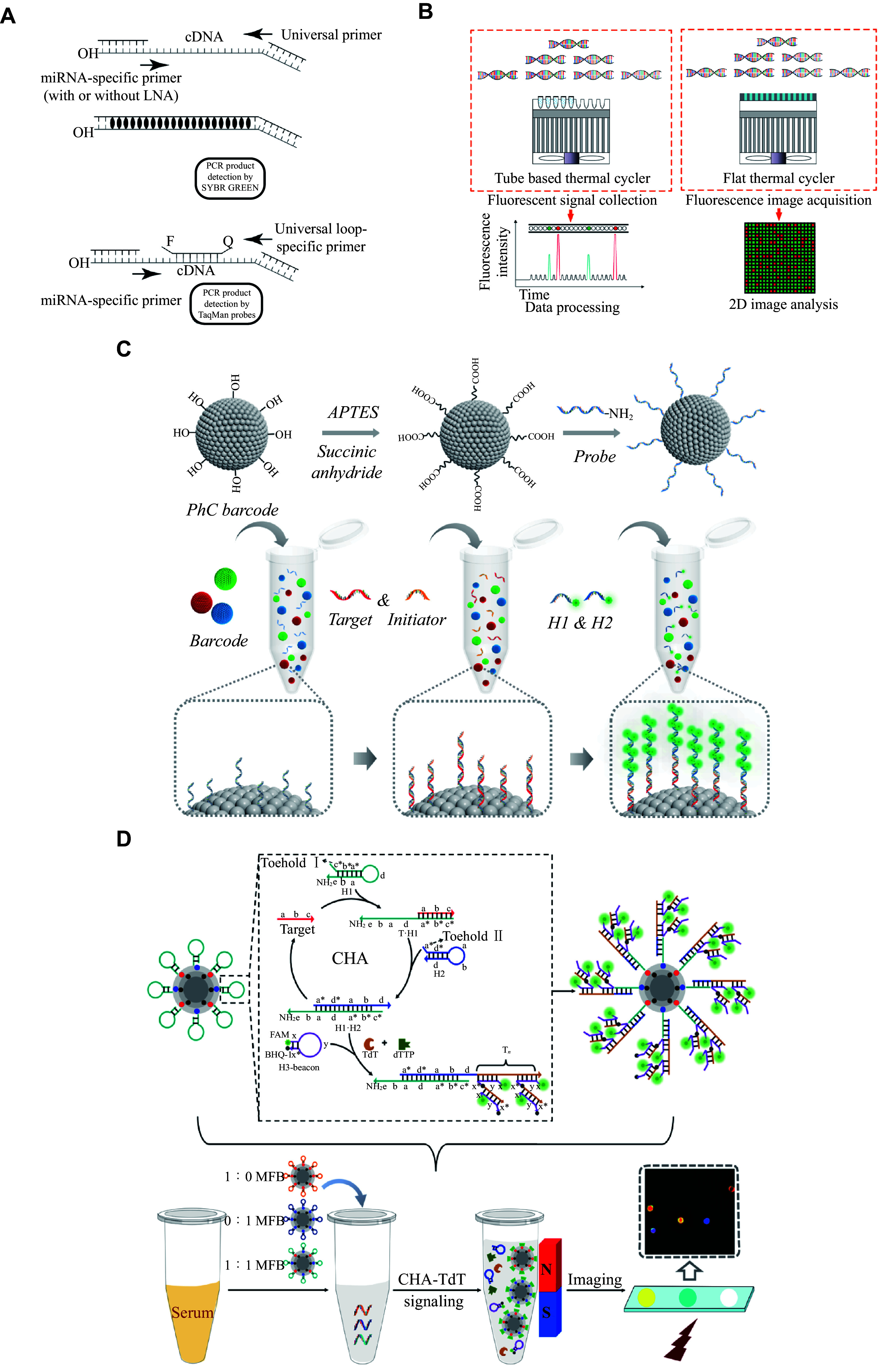
miRNA detection techniques.

An increasing number of studies based on the important biological roles of miRNAs and their close association with tumors have promoted the development of miRNA diagnostic techniques. However, the unique characteristics of miRNAs, such as small size, low content, and especially similar sequences of miRNA family members, also present challenges for laboratory detection^[93]^. The current reference method is RT-qPCR^[94]^ (***Fig. 4A***). Nevertheless, the complexities of sample preparation, such as RNA extraction, complex primer design, and high consumable costs, all of which limit its practical application^[95]^. In contrast to RT-qPCR, dPCR bypasses standardization and achieves absolute quantification^[96–97]^ (***Fig. 4B***). However, digital measurements still have some drawbacks, such as dynamic reaction process presentation or multiplexing capability^[98]^. Other traditional detection techniques, such as Northern blotting and microarrays, are time-consuming and insensitive to a certain extent^[99]^.

### Biosensing analysis

As a method based on principles such as electrochemistry or optics, biosensors are characterized by applicability, portability, low cost, and real-time analysis, and play an important role in miRNA detection^[100]^. Optimizing these bioassays involves specific signal sensors and uniquely designed biometric elements. Recently, the special physicochemical properties of nanomaterials and miRNA signal amplification strategies have been integrated effectively into the optimization of biosensors. Because of the enhanced molecular interactions, the diagnostic sensitivity of these biosensors continues to improve^[101–102]^ (***Fig. 4C*** and ***4D***). The biosensor-based assays combined with microfluidic devices for the point-of-care testing (POCT) analysis may achieve rapid, cost-effective, sensitive, and multiplexed detection of miRNAs^[103]^.

### NGS technology

As a breakthrough in the genome-wide assessment, NGS is used for high-throughput detection of both known and unknown miRNAs. It has the widest range of applications and has extremely high sensitivity and accuracy in distinguishing miRNAs with high sequence homology as well as alleles^[52,66]^. A recent study demonstrated that the synergistic effect of NGS technology with the surface-enhanced Raman spectroscopy (SERS) analysis of urinary miRNAs could better achieve POCT and molecular stratification of BC^[104]^. However, the professional data analysis and annotation capabilities to parse the specific functions of each dysregulated miRNA, as well as the high cost, have limited the use of NGS in traditional laboratories^[105]^.

### EV-derived miRNAs

**Figure 5:**
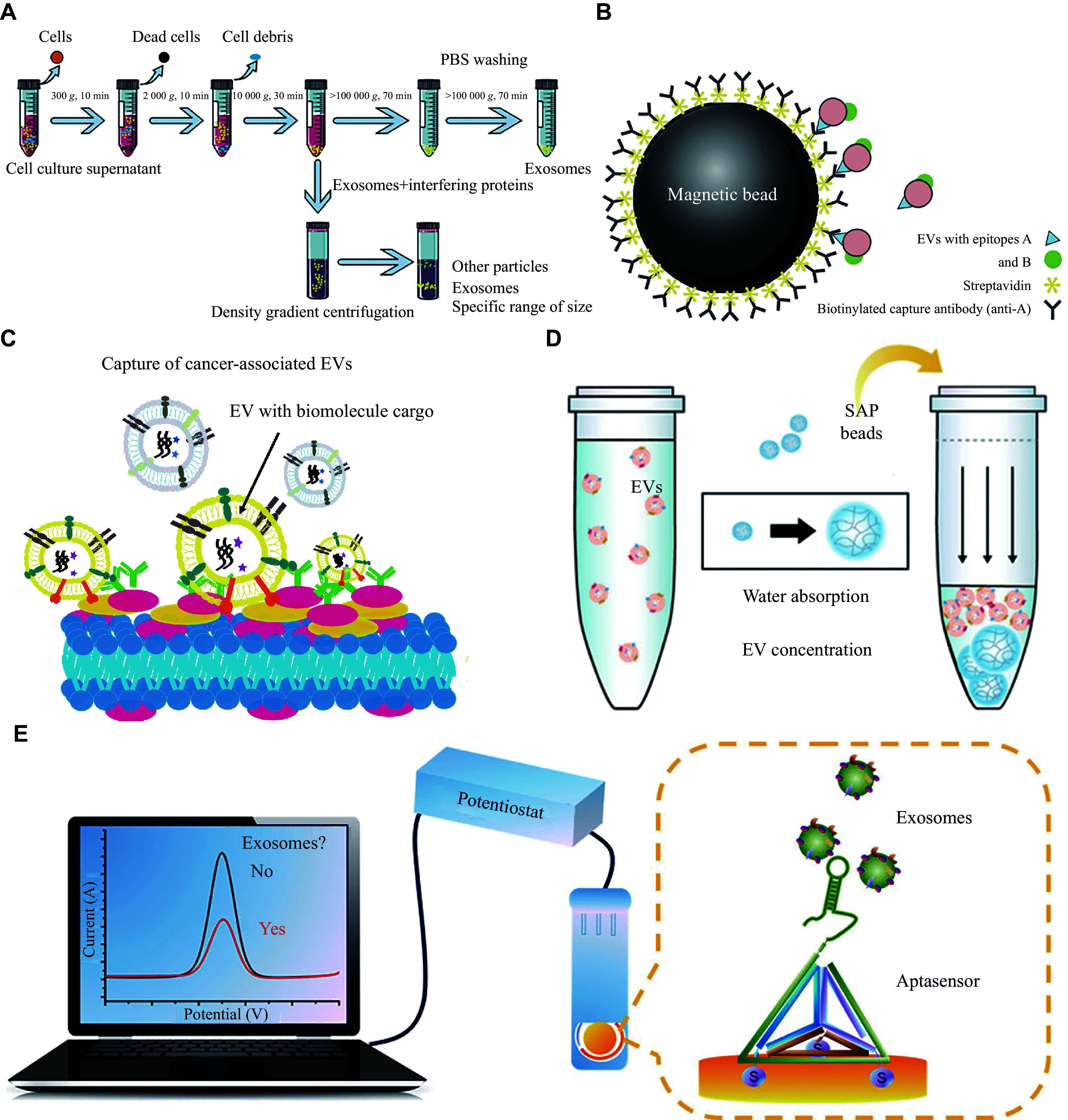
Extracellular vesicle (EV) extraction methods.

Investigation of the effect of EV miRNAs on the initiation and progression of BC holds a great potential. Nevertheless, the diminutive size, substantial heterogeneity, and prevalence in circulating body fluids, particularly in peripheral blood or other fluids with intricate compositions, pose a significant challenge in the isolation and identification of EVs^[106]^. The conventional techniques for separating particles, such as ultracentrifugation, density gradient centrifugation, and immunoaffinity purification, are characterized by intricate procedures, a limited capacity, an expensive implementation, and a prolonged duration, all of which inevitably lead to experimental inaccuracies^[107]^ (***Fig. 5A*** and ***5B***). The advancement of capture strategies that are both highly selective and efficient as well as avoiding the need for laborious purification steps, will facilitate the integration of liquid cancer biopsies based on EVs into routine clinical diagnostics^[108–109]^ (***Fig. 5C*** and ***5E***). Furthermore, to address the limitation of low abundance of EV-related biomarkers, the equipment-free concentration method that can enrich EVs in a simple step deserves further investigation^[110]^ (***Fig. 5D***). However, the identification of EV molecules as distinctive biomarkers has been infrequently suggested thus far, necessitating further comprehensive and profound investigations.

Taken together, each of the above-mentioned approaches has some advantages and disadvantages, which should be systematically considered, when they are used in developing research programs. Regardless of the choice, the evaluation of the analysis reliability needs to be compared with the laboratory reference method. Although extensive work has been done in miRNA research, a standardized and medically reliable method for miRNA detection and quantification is still needed to overcome the current technical deficiencies and challenges^[105]^.

## Challenges in the development of miRNAs as biomarkers and therapeutic targets

There are still some challenges in the translation of miRNAs as biomarkers and therapeutic targets into clinical practice. Firstly, BC has a high total mutational burden and considerable heterogeneity at genomic, transcriptional, and cellular levels, resulting in differences that remain difficult to quantify^[111]^. These significant differences are manifested among different sites of the same tumor, between primary and metastasis tumors, among different patients with the same type of cancer, and among different time points of tumorigenesis, progression, treatment, and recurrence. Secondly, inconsistencies in specimen source, collection and processing, and blood cell contamination in specimen preparation, as well as the lack of consensus on data normalization, make cross-platform comparisons difficult^[112]^. Thirdly, the specific post-transcriptional regulatory mechanisms and potential biological functions of miRNAs in BC are not particularly clear, and thus, comprehensive investigations and further validations for the clinical significance of miRNAs are needed. Fourthly, the unwanted toxicity, adverse reactions, and low efficacy of high doses involved in miRNA delivery technologies still need to be further studied^[113]^. Finally, the applications of miRNAs in BC therapy are still in their infancy, and no miRNA molecules have been designed and used in clinical trials of BC patients.

## Conclusions and perspectives

miRNAs are widely found in the blood, urine, and EVs, making them ideal candidates for non-invasive biopsies. By targeting related genes, miRNAs regulate cancer cell proliferation, colony formation, invasion, migration, metastasis, and chemotherapy resistance, thereby affecting the development and progression of BC. Dysregulated miRNA expression from samples of different BC patients may provide information about molecular basis of the tumors, and pave new ways for non-invasive diagnosis, prognostic monitoring, and the targeted therapy of BC.

Nevertheless, the existing literature presents inconsistent findings, likely attributed to analysis bias and specimen heterogeneity. Conducting extensive multicenter and prospective cohort studies involving the matched tissues and body fluids, along with the implementation of standardized specimen collection and laboratory diagnostic techniques, will significantly enhance the precision and dependability of diagnostic and prognostic outcomes. Furthermore, it is imperative to incorporate experimental controls, encompassing both benign and malignant tumors of the urinary tract as well as hematuria and hemolysis, to ascertain the diagnostic specificity of miRNAs for detecting BC. Moreover, the integration of diverse biomarkers and detection strategies in the multiplexed analysis of miRNAs holds a promising potential for advancing clinical applications.

Further investigations on the platforms for capturing EV sensitivity and identifying specific biomarkers will accelerate the advancement of novel biomarkers into clinical practice. Importantly, the integration of microfluidic technology and POCT in miRNA biosensors will establish a groundbreaking and efficient platform for the development of sensitive and simplified diagnostics for BC.

With the development of medical research, multidisciplinary collaboration is a great driving force for the advances, especially in oncology. Artificial intelligence (AI) serves as a notable illustration, as it enables the execution of various clinical tasks related to the diagnosis and prognosis of BC. These tasks encompass automated tumor detection, staging and grading, bladder wall segmentation, recurrence prediction, chemotherapy response monitoring, and survival assessment. Most AI-based diagnostic platforms are used in conjunction with cystoscopy, urine cytology, and imaging^[114]^. Given sufficient training data, potential genetic alterations will even be predicted from standard histopathological slides with accuracy comparable to molecular detection. We have sufficient reasons to believe that new insights into the application of miRNA in BC will be gained through the AI technology to analyze the interaction patterns of cellular phenotype and genomics^[115]^. Nevertheless, the possibility of excessive diagnosis and the complex mathematical characteristics of AI may hinder the capacity to employ analytical models in a comprehensive and comprehensible manner, thus requiring further collaborative investigation in the medical domain^[114]^.
